# Which Training Is More Effective in Post-COVID-19 Geriatric Patients with COPD: Cycle Ergometer Interval Training or Continuous Training?

**DOI:** 10.3390/life16020334

**Published:** 2026-02-14

**Authors:** Katarzyna Bogacz, Jacek Łuniewski, Anna Szczegielniak, Danuta Lietz-Kijak, Jan Szczegielniak

**Affiliations:** 1 Physiotherapy Department, Faculty of Physical Education and Physiotherapy, Opole University of Technology, Prószkowska 76, 45-758 Opole, Poland; 2 Ministry of Internal Affairs and Administration’s Specialist Hospital of St. John Paul II, Karłowicza 40, 48-340 Głuchołazy, Poland; 3 Stobrawskie Medical Center in Kup, Karola Miarki 14, 46-082 Kup, Poland; 4 Department of Psychoprophylaxis, Faculty of Medical Sciences in Zabrze, Medical University of Silesia, Poniatowskiego 15, 40-055 Katowice, Poland; 5 Department of Propaedeutics, Physical Diagnostics and Dental Physiotherapy, Pomeranian Medical University in Szczecin, Rybacka 1, 70-204 Szczecin, Poland

**Keywords:** COPD, COVID-19, physical exercise, interval training, continuous training, 6MWT

## Abstract

Introduction: Respiratory rehabilitation programs for geriatric patients with chronic obstructive pulmonary disease (COPD) after COVID-19 require a precise assessment of needs and an individualized approach. However, there is a lack of specific recommendations for aerobic training in this patient group. Objective: The study aimed to compare two types of aerobic training—continuous and interval—and to determine which one is more effective and should be included in the respiratory rehabilitation program for geriatric patients with COPD after COVID-19. Methods: Of the 480 patients examined, 80 were included in the study. All patients underwent exercise tolerance tests (6-Minute Walk Test—6MWT) and functional performance tests (get-up-and-go test—TUG) before and after a 3-week intensive respiratory rehabilitation program. Results: Both types of training—interval and continuous—contributed to improved exercise tolerance and functional fitness in patients. However, analysis of the differences between the groups showed that continuous training with increasing exercise intensity resulted in significantly greater improvements in distance covered during the 6MWT, energy expenditure (METs), and TUG test time (*p* < 0.05). Conclusions: Continuous training on a cycle ergometer is more effective in the rehabilitation of geriatric patients with COPD after COVID-19 and should be included in therapeutic programs.

## 1. Introduction

Chronic obstructive pulmonary disease (COPD) in older individuals is characterized by chronic airway inflammation and alveolar destruction, leading to dyspnea, cough, and sputum production, with exacerbations of varying intensity caused by respiratory tract infections [1]. Initial reports from the Centers for Disease Control and Prevention indicated that COVID-19 disproportionately affects patients with COPD, likely due to the presence of other comorbidities [2]. Studies examining the relationship between COPD and COVID-19 have shown that COPD patients experience worse outcomes when infected with COVID-19, although the specific mechanisms remain unclear [3]. It has been hypothesized that factors such as severe inflammatory responses, genetic susceptibility, and the aging immune system may play a significant role in the clinical picture and management of COPD patients with COVID-19 [4].

Due to nonspecific symptoms and limited use of lung function tests in daily practice, the prevalence of COPD among older adults is probably underestimated [5]. Research shows that patients with COPD experience greater participation restrictions—meaning functional limitations in performing expected tasks in physical and sociocultural settings—compared to age-matched individuals without respiratory conditions [6]. COPD negatively affects quality of life, with symptom severity and exacerbation frequency being major predictors of mental health issues, such as depression and anxiety, which can further worsen overall health [7].

Numerous articles on pulmonary rehabilitation programming for patients with respiratory diseases emphasize the importance of aerobic, anaerobic, and breathing exercises. However, there are no specific guidelines for aerobic training, although most programs incorporate a fixed duration and gradual intensity build-up [8]. Published works typically discuss duration, heart rate limits, and intensity levels to be reached during exercise [9,10,11,12,13,14,15,16].

Systematic physical exercise of appropriate intensity, frequency, and duration has been shown to improve lung ventilation, chest mobility, exercise tolerance, and overall physical condition, thereby enhancing subjective well-being in patients [17,18,19,20,21]. Pulmonary rehabilitation in geriatric patients reduces disease exacerbations, hospitalizations, and psychosocial dysfunction, and improves quality of life [22,23,24,25,26]. Implementing complex therapy, including patient qualification based on exercise tests, spirometry results, dyspnea assessment, and physical function assessment, significantly benefits patients [27,28,29,30].

Pulmonary rehabilitation for COPD patients, understood as a comprehensive and individualized therapeutic intervention aimed at improving physical and mental health, should include supervised physical exercises at least twice a week (preferably three times weekly for optimal results) for 6–8 weeks. However, data on which exercise modality is most beneficial are still lacking. Exercise intensity should be appropriately adapted to patient capacity and modified based on progress; simultaneously, it should exceed the loads required for daily activities [31]. Recommendations from the American College of Chest Physicians and the American Association of Cardiovascular and Pulmonary Rehabilitation emphasize that upper- and lower-extremity training, patient education, and resistance training are core elements of pulmonary rehabilitation [32]. The 2017 European Respiratory Society and American Thoracic Society guidelines highlight that while pulmonary rehabilitation within 3 weeks after hospital discharge following a COPD exacerbation reduces readmissions and improves quality of life, as well as implementation within 8 weeks improves exercise capacity, specific interventions yielding the most significant benefits remain to be determined [33]. Scientific evidence also suggests that nonlinear periodized exercise training may benefit some COPD patients, offering an alternative approach that adapts to individual needs [34].

Based on these considerations, the current study aimed to compare two types of aerobic training—continuous and interval—and identify which one is more effective for inclusion in pulmonary rehabilitation programs for elderly patients with COPD after COVID-19.

.

## 2. Materials and Methods

### 2.1. Participants

A total of 480 patients participated in the post-COVID-19 pulmonary rehabilitation program. Patients were classified into models A, B, C, D and E based on an assessment of exercise parameters such as the 6-Minute Walk Test (6MWT) score, FEV1 value and dyspnea level according to the Borg scale.

Patients classified as model C were chosen for the study because of their moderate exercise tolerance (3–5 METs on the 6MWT) and FEV1 values of 30–50% of predicted, indicating an intermediate disease stage. This level of severity allowed for the safe use of aerobic training protocols and resulted in varied bodily responses to rehabilitation efforts, making these patients ideal for assessing both continuous and interval training effectiveness. 

At the end, research involved 80 patients (46 females and 34 males) diagnosed with COPD, up to 6 months after COVID-19, who were admitted for medical treatment at the Ministry of Internal Affairs and Administration’s Specialist Hospital of St. John Paul II in Głuchołazy between December 2021 and May 2022. The average age of the patients was 76 years (SD = 6.20) [Table 1]. There is no single age cutoff for elderly patients, but most definitions of geriatric medicine include individuals aged 65 or 70 and older. For this study, we adopted an age limit of 70 years. Patients participated in the hospital’s standard rehabilitation program.

Inclusion criteria consisted of: age ≥ 70 years, diagnose of COPD by pulmonary specialist (cut off values: below 80% of FEV1 (Forced Expiratory Volume in 1 s) predicted and FEV1%VC (Forced Expiratory Volume in one second% of Vital Capacity) below lower limit of the norm, lack of significant improvement in reversibility), in stable condition (no exacerbations), referred to physiotherapy, with patient’s informed consent to participate in the study obtained, classified for model C of rehabilitation. Exclusion criteria included: acute stage of respiratory disease, comorbid diseases significantly influencing the patient’s exercise tolerance, musculoskeletal system diseases significantly affecting mobility, non-standard pharmacological treatment, and lack of informed consent to participate in the study.

### 2.2. Interventions

All patients included in the research underwent exercise tolerance and physical function tests before and after the 3-week intensive physiotherapy.

Exercise tolerance was assessed in the 6-Minute Walk Test. The test was conducted before and after the pulmonary rehabilitation program. Before the program the test was administered twice. The first was the control test, and the second, given 24 h later, was the proper test. Before and after the test, each patient had their blood pressure and heart rate taken, and their dyspnea level was assessed through the 0–10 fixed point scale. The patients were informed about the aim of the test and procedure. They were also instructed about the obligation to abstain from cigarettes, coffee, and alcohol before the test. They were also advised to take a 15 min rest before the test. The energy expenditure level was calculated based on the distance covered in the test and the walking speed. The energy expenditure index was expressed in MET (Metabolic Equivalent of Task) units following the methodology described in the work by Szczegielniak et al. [28].

The patient’s functional capacity was assessed in the TUG test. This test aimed to determine the patient’s mobility as well as dynamic balance. The test began with the patient sitting on a chair. The patient’s hands rested on their laps, feet placed flat on the floor. A cone was placed 2.44 m away from the chair. On the “start” command, the patient was supposed to stand up from the chair, walk the marked distance, go around the cone, return, and sit on the chair in the initial position. The task was timed from the “start” command, irrespective of whether the patient reacted immediately or with delay until the initial sitting position on the chair was achieved. The measure was taken accurately at 0.1 s. The best time achieved in two tries represented the test result [27,35].

All patients followed the same study protocol. Individual exercise loads were determined for each patient. The program in both groups included breathing exercises (one 30 min session per day, 6 times a week), general physical function training (one 30 min session per day, 6 times a week), cycle ergometer training (one 30-minute session per day, 6 times a week), resistance training (one 30 min session per day, 3 times a week), circuit training (one 30 min session per day, 3 times a week), walking (one 30 min session per day, 6 times a week), inhalations with 0.9% NaCl solution (one 15 min session per day, 6 times a week), postural drainage and chest clapping in the lying position on the back, on the right side, and on the left side (one 15 min session per day, 6 times a week). The exercise intensity was set at 60% of submaximal heart rate. (Table 2).

### 2.3. Study Groups and Cycle Ergometer Trainings’ Protocoles

Patients were randomly assigned to training groups through simple randomization and were also systematically recorded during their training sessions, enabling ongoing assessment of compliance with the study protocol.

The continuous training group consisted of 40 patients (22 females and 18 males). The average age of the patients was 75 years old (SD = 5.35). The interval training group consisted of 40 patients (24 females and 16 males). The average age of the patients was 77 years old (SD = 4.05) (Table 1). Patients in the continuous training group were presented with the continuous cycle ergometer training program with increasing exercise load. Training started with a 40 W load applied for 4 min. Next, the load was increased by 10 W, and the exercise continued until the training heart rate limit was reached. Then, the training continued at a stable intensity at the training heart rate limit for 30 min per participant. Patients in the interval-training group were presented with progressively increasing exercise loads. Training began with a 40 W load and lasted 4 min. A 2-min break followed this. For the second cycle, the exercise load was increased by 10 W, and exercise continued until the training heart rate limit was reached. The exercise workload was not increased further in subsequent cycles. One training session included between three and six 4 min cycles and lasted 30 min for each participant.

### 2.4. Statistical Analysis

The collected data were processed via the Statistica 13.3 program (StatSoft, Inc., Chicago, IL, USA), licensed by the Opole University of Technology. The Kolmogorov–Smirnov test was used to assess the normality of distributions, and the results indicate that the assumptions of normality for the analyzed variables are satisfied. The results of the Kolmogorov–Smirnov test were as follows: for the 6MWT distance variable D = 0.093, *p* = 0.20; for the MET energy expenditure variable D = 0.085, *p* = 0.15; and for the TUG time D = 0.078, *p* = 0.10. Therefore, the normality assumption was confirmed for all variables, justifying the use of Student’s *t*-test to evaluate intra- and intergroup differences. A significance level of *p* < 0.05 was adopted for all variables.

## 3. Results

Results for both continuous and interval training showed significant improvement in the assessed treatment outcomes. All variables showed statistically significant differences in values measured before and after the therapeutic program was introduced.

In the continuous training group, the distance variable in the 6MWT showed higher mean values after therapeutic program, and the energy expenditure variable expressed in MET assessed in the 6MWT showed higher mean values than before therapeutic program (*p* < 0.05).

In the interval training group, the distance variable in the 6MWT showed higher mean values after the therapeutic program, and the energy expenditure variable expressed in MET assessed in the 6MWT showed higher mean values than before therapeutic program (*p* < 0.05). A comparison of the distance variable and the energy expenditure variable expressed in MET assessed in the 6MWT and basic descriptive measures, are presented in Table 3.

The differences between groups were analyzed to assess which training is more beneficial for the patients. Results of Student’s *t*-test for 6MWT (in meters) indicate that the difference between pre- and post-training values in the continuous training group is greater than in the interval training group. Statistical significance was established at *p* < 0.05 (t = 11.393, df = 39, *p*-value = 5.612 × 10^−14^). This indicates a bigger improvement in distance after physiotherapy in the continuous training group. Results of Student’s *t*-test for 6MWT (in MET) show that differences in the values before and after in the continuous training group are higher than in a group with interval training. Statistical significance was established at *p* < 0.05 (t = 9.1006, df = 39, *p*-value = 3.441 × 10^−11^). This indicates a bigger improvement in energy expenditure expressed in MET after physiotherapy in the continuous training group (Table 3).

The observed 20 m difference in 6MWT test results before rehabilitation between the groups is due to natural variability within the sample, despite random assignment. Although this difference exists, proper randomization was performed, and it does not affect the interpretation of the therapeutic effects.

Analysis conducted with Student’s *t*-test showed that the mean values in the functional capacity test after therapeutic program were significantly higher for both groups.

Analysis of differences in functional capacity between the two groups after completing the therapeutic program was conducted. Results of Student’s *t*-test point out that differences in the values in the continuous training group are higher than those in the interval training group. Statistical significance was established at *p* < 0.05 (t = −10.961, df = 39, *p*-value = 1.789 × 10^−13^). This indicates a bigger improvement in functional capacity after therapeutic program in the continuous training group (Table 4).

## 4. Discussion

Aerobic training is a form of physical exercise that increases heart and breathing rates to enhance cardiovascular fitness and endurance. It can be performed as interval or continuous training with a fixed or gradually increasing exercise load [35,36,37,38,39]. Although there are various options for aerobic training modes, the optimal intensity or personalized exercise strategy for respiratory disease patients with respiratory issues remains unclear. Practitioners have already discussed practical considerations for using interval training in respiratory patients in the literature [40]. However, these references pertain to a different form of interval exercise than what is used in this study. The training involves 30 s of exercise followed by 30 s of rest, or 20 s of exercise followed by 40 s of rest, starting at an intensity of 80–100% of peak work rate (PWR) for the first three to four sessions. Afterwards, the workload is increased by 5–10% as tolerated, gradually reaching 150% of the baseline PWR. Additionally, the ATS/ACCP recommends the development and validation of new exercise protocols for specific indications [41].

This study assessed exercise intensity after a prior qualification process for well-defined rehabilitation models. As the results indicate, interval and continuous training, when used as part of a comprehensive therapeutic program for geriatric patients with respiratory disease, enhanced exercise tolerance and physical function. In the group that received continuous training, significantly greater improvements in physical function (TUG) were observed, along with higher gains in distance and MET during the 6MWT. In the publication Gloecki et al. [40], it was also shown that exercise significantly improved physical fitness tolerance.

There is evidence that, in patients with very severe COPD, interval training is associated with reduced breathlessness during exercise. Alternating periods of activity and rest helps patients decrease respiratory muscle fatigue. Studies show that this method prevents excessive strain on the respiratory system, which is often a problem with continuous training in advanced COPD [42]. Additionally, compared to continuous training, interval training is viewed as more comfortable for patients with severe shortness of breath, which may boost their motivation to stick with therapy. Including interval training in pulmonary rehabilitation programs for people with COPD could lead to better management of shortness of breath and a higher quality of life. However, further research is required to compare the long-term effects of interval and continuous training on respiratory function and overall physical performance in COPD patients [43].

A systematic review of eight randomized controlled trials (RCTs) involving 388 patients with COPD found that interval and continuous training modalities did not differ in their effects on exercise capacity measures or health-related quality of life (HRQoL) [44]. A 2021 meta-analysis, which included 13 RCTs (11 with COPD patients), incorporated five additional studies beyond previous reviews, providing an updated synthesis of the evidence. The main conclusions concerned patients with chronic respiratory diseases (CRD), but were strongly dominated by data from COPD. Interval exercise training (IET) was found to be statistically superior to continuous exercise training (CET) in terms of improvement [45]. Systematic reviews provide somewhat inconsistent but generally convergent conclusions. Both interval and continuous training are effective in improving exercise capacity, symptoms, and quality of life in patients with COPD [44,45]. IET is statistically superior to CET in improving peak power and reducing exercise-induced breathlessness, but this advantage is not clinically significant [44,45]. IET is the preferred training option for patients with CRD (including COPD) who are unable to maintain continuous exercise due to severe exertional dyspnea and arterial hypoxemia [44,45]. One study included in the reviews suggests that IET may lead to greater reductions in oxygen cost and ventilation at submaximal exercise (isotime) compared to CET [46]. Overall, although early reviews showed no differences, more recent data suggest a small, statistically significant advantage of IET over CET in improving exercise capacity and reducing dyspnea, particularly in patients with COPD and obesity. IET is a valuable modality because it allows patients to exercise at a higher intensity than continuous training, which is crucial for physiological adaptations, especially for patients with severe ventilation limitations.

The results prove that aerobic training preceded by adequate qualification and properly calculated exercise load improves exercise tolerance and physical function. This was reflected in the increase in distance and energy expenditure parameters in the 6MWT and the decrease in the time parameter in the TUG test. Therapeutic program that consists of aerobic training with cycle ergometer, both in continuous and interval modes, is effective for geriatric patients with COPD. Nevertheless, a significantly higher improvement was observed among the group that used continuous training with increasing exercise intensity. This might result from the fact that continuous training methods primarily improve aerobic capacity and increase the efficiency of cardiovascular and respiratory systems. They are also the basic tool for developing both general and directed endurance.

Therapeutic programs for geriatric patients with COPD after COVID-19 require precision, need assessment, and an individualized approach [47,48,49]. Providing tailored treatment in a hospital environment may be challenging as the group is not homogeneous in terms of general health condition, comorbidities, and long COVID-19 symptoms. There is a strong need for additional evidence on risk profiles of older patients living with COPD to develop individualized treatment regimes. Respiratory muscle weakness in older people can increase the incidence of disease and disability due to reduced lung flexibility, respiratory muscle strength, and chest wall compliance [50]. To date, several different exercises have been proposed to improve the fitness of older people, including Respiratory Muscle Training (RMT). It results in stronger diaphragm function and improved aerobic and cough capacities. It should be noted that in people with chronic respiratory diseases, there is a secondary impairment of respiratory muscle function, which is exacerbated by the persistence of systemic inflammation, the effects of the drug treatment used, and the reduced physical activity of these patients [51,52]. In a 2021 study, COVID-19 patients showed poor mechanical performance (impaired breathing capacity, leg discomfort, and peripheral muscle dysfunction) that was similar to that seen in patients with COPD. COVID-19 infection may be an additional factor that adds to age and COPD symptoms by limiting exercise tolerance [53]. Although respiratory muscle training is included in pulmonary rehabilitation, using inspiratory muscle training as an essential component is not always indicated. A paper comparing short-term inspiratory resistance training (R-IMT) and inspiratory threshold training (T-IMT) in patients with COPD showed that improved exercise tolerance was associated with increased inspiratory muscle reserve in both R-IMT and T-IMT. Still, R-IMT was associated with deeper, slower breathing and improved ventilatory efficiency [54]. The 2021 study showed that there is a need to assess the threshold levels of inspiratory muscle strength below which exercise capacity is impaired in people with COPD [55]. From the perspective of pulmonary rehabilitation as a holistic process, it is also worth noting that in older patients there are changes in gait mechanics and energy costs, as well as changes in connective tissue and neuromuscular conduction [56].

Both the benefits and the effectiveness of continuous and interval training depend on individual goals and fitness level. Continuous training, also known as steady-state training, involves performing cardiovascular exercise at a constant intensity for an extended period. It improves cardiovascular endurance and performance in endurance sports. Conversely, interval training involves alternating periods of high-intensity exercise with periods of low-intensity or rest. It improves cardiovascular fitness, speed, and power, but may be too burdensome for older people. Therefore, we recommend continuous training in geriatric patients with COPD after COVID-19.

Although the results show the effectiveness of both types of training, the study has several significant limitations. It was conducted at a single center with a relatively small number of participants, which may limit the extent to which the results can be generalized. Additionally, the absence of long-term follow-up and the homogeneity of the study group (patients with moderate COPD) may influence the interpretation of the durability of the effects and their relevance to a broader patient population. Nonetheless, the findings offer valuable insights into the potential benefits of both training methods in geriatric rehabilitation.

## 5. Conclusions

All elements of the therapeutic program contribute to the observed improvement. Both individualized interval training and continuous training on cycle ergometer as a part of a complex pulmonary rehabilitation program for geriatric patients with COPD after COVID-19 lead to improvement in exercise tolerance and functional capacity. However, continuous training is more effective for this patients and, thus, recommended for inclusion in the therapeutic programs. Further research is needed to understand the long-term effects of COVID-19 on the body and the recovery process to develop guidelines for specific patient groups.

## Figures and Tables

**Table 1:** Characteristics of participants.

	All Patients N = 80	Continuous Training N = 40	Interval Training N = 40
Gender, n (F/M)	46/34	22/18	24/16
Age, mean (SD)	76 (6.20)	75 (5.35)	77 (4.05)
FEV1 mean (SD)	73.45 (7.76)	71.25 (8.65)	74. 65 (6.54)
FEV1%VC	56.81 (1.78)	55.75 (2.47)	57.52 (1.84)

**Table 2:** Pulmonary rehabilitation model C by J. Szczegielniak.

Model C of Pulmonary Rehabilitation	Duration per Session	Frequency per Week	Details
Breathing exercises	15 min	6 times	Diaphragmatic and pursed-lip breathing techniques.
General physical function training	30 min	6 times	Stretching exercises, balance exercises, and coordination exercises.
Cycle ergometer training	30 min	6 times	Endurance training up to 60% of the submaximal heart rate.
Resistance training	30 min	3 times	Exercises using weights and resistance bands.
Circuit training	30 min	3 times	Strength, endurance, and flexibility exercises.
Walking	30 min	6 times	Moderate-intensity walking, adjusted to patients.
Inhalations with 0.9% nacl solution	15 min	6 times	Used for airway hydration and clearing of retained secretions.
Postural drainage nad chest clapping	15 min	6 times	To facilitate mucus clearance in a lying position on the back, right, and left sides, with chest percussion in the same positions.

**Table 3:** Variable distance and MET in the 6MWT, before and after therapeutic program.

**Measure**	**Continuous Training** **Before Physiotherapy**	**Continuous Training** **After Physiotherapy**	**Interval Training** **Before Physiotherapy**	**Interval Training** **After Physiotherapy**	**Between-Group Differences** **After Physiotherapy**
Average Distance [m]	401.13	475.73	420.05	447.83	-
Standard Deviation (Distance)	35.23	39.73	28.25	30.65	-
Difference (Distance)	-	74.60	-	27.78	27.9
T-tests (Distance)	-	14.966	-	12.818	11.393
*p*-value (Distance)	-	*p* < 0.05	-	*p* < 0.05	*p* < 0.05
Average Energy Expenditure [MET]	4.332	5.945	4.701	5.304	-
Standard Deviation (MET)	0.69	0.93	0.55	0.65	-
Difference (MET)	-	1.613	-	0.603	0.641
T-tests (MET)	-	12.43	-	10.514	9.1006
*p*-value (MET)	-	*p* < 0.05	-	*p* < 0.05	*p* < 0.05

Student’s *t*-test established a statistical significance level at *p* < 0.05 for the functional capacity variable. Obtained values prove that all measures show statistically significant differences.

**Table 4:** Physical function, assessed in the TUG test, before and after therapeutic program.

Physical Function Assessed in the TUG Test	Continuous Training Before Physiotherapy	Continuous Training After Physiotherapy	Interval Training Before Physiotherapy	Interval Training After Physiotherapy	Analysis of Between-Group Differences After Physiotherapy
Average Time [s]	17.850	10.700	17.500	14.550	-
Standard Deviation	3.853	3.458	4.032	3.336	-
Difference	-	7.150	-	2.950	3.850
T-tests	-	17.062	-	8.500	10.961
*p*-value	-	*p* < 0.05	-	*p* < 0.05	*p* < 0.05

## Data Availability

The data that support the findings of this study are not openly available due to reasons of sensitivity and are available from the corresponding author upon reasonable request.

## Abbreviations

6MWT	6-Minute Walk Test
COPD	Chronic obstructive pulmonary disease
FEV1	Forced Expiratory Volume in 1 s
FEV1%VC	Forced Expiratory Volume in one second% of Vital Capacity
TUG	Timed Up and Go
MET	Metabolic Equivalent of Task
